# Renal replacement therapy in sarcoidosis

**DOI:** 10.3389/fmed.2022.990252

**Published:** 2023-01-09

**Authors:** Marta Calatroni, Gabriella Moroni, Claudio Ponticelli

**Affiliations:** ^1^Department of Biomedical Sciences, Humanitas University, Milan, Italy; ^2^Nephrology and Dialysis Division, IRCCS Humanitas Research Hospital, Milan, Italy; ^3^Independent Researcher, Milan, Italy

**Keywords:** end-stage kidney disease, renal replacement therapy, granulomatous interstitial nephritis, hypercalcemia, nephrocalcinosis, renal transplantation

## Abstract

Sarcoidosis is a systemic inflammatory disease of unknown etiology. Kidney involvement in sarcoidosis may be present in up 25–30% of cases. An early diagnosis and prompt treatment with corticosteroids can improve the prognosis but rarely renal sarcoidosis can lead to kidney failure needing renal replacement therapy (RRT). Acute kidney injury (AKI) in sarcoidosis may be caused by granulomatous interstitial nephritis (GIN) or hypercalcemia. These disorders are usually clinically silent and may lead end stage renal disease (ESKD) if not diagnosed or detected too late. In patients with ESKD, dialysis and renal transplantation can offer results comparable to those observed in patients with other causes of kidney failure. Based on a review of literature, we present an overview of RRT in patients with AKI or chronic kidney disease (CKD) caused by sarcoidosis.

## Introduction

Sarcoidosis is an inflammatory systemic disease of unknown etiology characterized by abnormal collections of inflammatory cells that form granulomas. Sarcoidosis usually occurs between the ages of 20 and 50 years and women are 30% more affected than men (1). The disease occurs worldwide with an estimated prevalence of 10–160 per 100,000 population. However, there are significant differences in the various geographical regions of the world with higher prevalence in Afro-Americans, Caribbean, and in Northern countries (2–7). The diagnosis of sarcoidosis is difficult and the disease may remain undetected in several cases. In the absence of standardized criteria, a panel of the American Thoracic Society proposed to use three major criteria: a compatible clinical presentation, finding non-necrotizing granulomatous inflammation in one or more tissue samples, and the exclusion of alternative causes of granulomatous disease (8). Sarcoidosis may affect multiple organs. The lungs are the affected organ in >90% of cases and pulmonary sarcoidosis may be responsible of progressive respiratory disease, that remains a leading cause of death and disability (9, 10). Renal involvement in patients with sarcoidosis occurs in up to 25–43% (11, 12). A wide spectrum of renal diseases has been reported in patients with sarcoidosis, including granulomatous interstitial nephritis (GIN), several subtypes of glomerulonephritis, renal stones and nephrocalcinosis (13–15) (Table 1). Kidney involvement may be severe, leading to kidney failure in acute and in chronic disease. Only case reports and few small retrospective studies have been reported in literature and little information is available about treatment and prognosis of patients with kidney failure. Notwithstanding, an early recognition of these diseases is important to start early the treatment and to establish the prognosis. In this descriptive review, we will consider the available data on cases of acute kidney injury (AKI) and chronic kidney disease (CKD) in sarcoidosis, requiring renal replacement therapy (RRT).

**TABLE 1:** Main renal diseases reported in patients with sarcoidosis.

Granulomatous interstitial nephritis
Glomerular diseases
FSGS Mesangial proliferative GN
IgA nephropathy
Membranoproliferative GN
Membranous nephropathy
Crescentic GN
Hypercalcemia
AKI
Nephrolithiasis
Nephrocalcinosis

FSGS, focal segmental glomerulosclerosis; GN, glomerulonephritis; AKI, acute kidney injury.

## Materials and methods

We conducted a literature search from ’70s to May 2022 in PubMed, Medline, and Embase, and from reference list of retrieved articles. During searching, we used these terms and keywords: renal sarcoidosis, acute kidney injury, chronic kidney disease, renal replacement therapy, granulomatous interstitial nephritis, hypercalcemia, nephrocalcinosis, and renal transplantation. Study quality and recommendations were assessed based on importance of the published studies.

### Acute dialysis

Severe acute renal failure requiring dialysis may occur in sarcoidosis. It is mainly due to granulomatous interstitial nephritis, hypercalcemia, and/or hypergammaglobulinemia. Many patients with renal sarcoidosis are affected by non-caseating GIN, a disorder that responds to corticosteroids and immunosuppressive drugs. Clinically, GIN may be silent or may present with abnormal urine analysis such as microscopic hematuria, mild proteinuria or sterile leukocyturia sometimes associated with high serum creatinine levels and low glomerular filtration rate (GFR). GIN may also be severe and lead to AKI and requiring dialysis (16–23). In some cases, AKI is the first manifestation of sarcoidosis (24, 25). In a retrospective study on 12 cases of sarcoidosis complicated by AKI renal biopsies showed tubule-interstitial nephritis in all patients, with granuloma in six patients and giant cells in two cases. Five patients (40%) needed hemodialysis for severe renal impairment. All patients received prednisone 1 mg/kg/day with gradual improvement of renal function, normalization of serum creatinine levels and no renal recurrence of AKI after 5 years (16).

The differential diagnosis between AKI due to granulomatous interstitial nephritis or other diseases may be difficult without renal biopsy. Cases of AKI caused by IgA nephritis (26) or associated with elevated levels of anti-glomerular basement antibodies (27) in patients with sarcoidosis have been reported. In addition, cases of sarcoidosis with ANCA-positive crescentic glomerulonephritis have been described (28). Prompt diagnosis and treatment may result in partial or complete recovery but late diagnosis can result in irreversible kidney damage. Another cause of AKI is represented by hypercalcemia. It is defined by a total serum calcium concentration >10.4 mg/dl (>2.60 mmol/L) or ionized serum calcium >5.2 mg/dl (>1.30 mmol/L). Depending on the population studied, about 2–63% of sarcoidosis patients show hypercalcemia (29). Elevated levels of serum calcium are probably due to overproduction of activated polarized macrophages expressing very high levels of the 25(OH)D-1α-hydroxylase that convert 25(OH)D into 1,25(OH)_2_D_3_ (30). Mild hypercalcemia (<11.5 mg/dl) is often asymptomatic or may be associated with aspecific gastrointestinal troubles. An acute increase in serum calcium over 12 mg/dl (3.00 mmol/L) may lead to AKI through different mechanisms (i) hypercalcemia causes intracellular calcium overload and tubular obstruction by calcium precipitates (31, 32), (ii) hypercalcemia increases renal vascular resistance and reduces GFR (33), (iii) hypercalcemia induces renal resistance to vasopressin leading to polyuria, dehydration, and hypovolemia (34). Severe hypercalcemia (>14 up to 18 mg/dl) can cause neuromuscular symptoms, hyporeflexia, dehydration, confusion, and acute renal failure. A shortened QTc interval is shown on electrocardiogram and arrhythmias may occur. Acute hypercalcemia is a rare event in sarcoidosis, but several cases of hypercalcemia-related acute renal failure have been reported (35–41). In a few cases, the concomitance of hypercalcemia and acute renal failure may be caused by peritoneal sclerosis (42). Hypercalcemia is often asymptomatic and may remain undetected if it is not measured routinely. Hypercalciuria is even more frequent. It is often associated with interstitial inflammation and may predict a good response to immunosuppressive therapy (43). Treatment of hypercalcemia depends on the severity of the disorder. Mild hypercalcemia can be managed with corticosteroids, thiazide diuretics, hydroxychloroquine, or ketoconazole. In intermediate cases (serum calcium between 12 and 14 mg/dl) corticosteroids, bisphosphonates, or denosumab are indicated. Severe hypercalcemia is a life-threatening complication and needs an emergency treatment. The more effective and safest management consists in dialysis. If dialysis is not possible saline infusion, calcitonin and intravenous bisphosphonates, and denosumab are indicated. Some patients with sarcoidosis and myeloma may present AKI, associated with hypergammaglobulinemia, normal albuminemia and hypercalcemia. In these cases, the differential diagnosis between sarcoidosis and multiple myeloma may be difficult (44, 45). Bone marrow biopsy, showing non-caseating granulomas or prompt improvement of hypercalcemia and kidney function after corticosteroid administration may lead to a correct diagnosis (46).

### Regular dialysis

Granulomatous interstitial nephritis is often clinically silent and but may be complicated and lead to end stage kidney disease (ESKD) if not diagnosed or detected too late. Sarcoidosis-associated glomerular diseases, including focal and segmental glomerulosclerosis (47), IgA nephropathy (48), and membranous nephropathy (49) may also progress to ESKD. In a retrospective study of 2009, The French Sarcoidosis Group described 47 adults with renal sarcoidosis and reported that more than 60% of them had a GFR <60 ml/min per 1.73 m^2^ and two patients needed hemodialysis after a median follow-up of 24 months (50). In that study the long-term renal function was inversely related to the initial fibrosis score at kidney biopsy and patients with an extensive interstitial fibrosis were unresponsive to treatment. Similar results have been found by Rajakariar et al. (51) and by Berliner et al. (52). In the past, the most frequent cause of CKD in sarcoidosis was nephrocalcinosis, a disorder characterized by the deposition of calcium salts in the kidney parenchyma and tubules, that may lead silently to ESKD (53–56). More recently, a systematic renal screening in sarcoidosis, including instrumental diagnosis with abdominal x ray, ultrasonography and/or computed tomography, allowed to detect and manage silent stones and calcium deposits in the kidney and to prevent their progression to ESKD. However, a small number of patients with long-term asymptomatic hypercalcemia and hypercalciuria may still develop nephrocalcinosis and require RRT (57–59). In many instances, patients needing regular dialysis for unknown causes develop asymptomatic or oligosymptomatic hypercalcemia which reveal the presence of an underlying sarcoidosis (60, 61). Both periodic hemodialysis and continuous ambulatory peritoneal dialysis have been successfully used in patients with uremic sarcoidosis. However, peritoneal dialysis can be ineffective in patients with peritoneal sarcoidosis, a possible, although unusual, presentation of the disease (62–64). A main issue in sarcoidotic patients on regular dialysis is represented by hypercalcemia. In uremic patients, there is usually hyperphosphatemia, hypocalcemia, and the poor kidney conversion from 25(OH) to the active 1,25(OH)_2_D_3_**.** These abnormalities stimulate an excessive production of parathyroid hormone (PTH) leading to secondary hyperparathyroidism and progressive development of uremic osteodystrophy, characterized by osteitis fibrosa, and a high turnover bone disease (65). A decreased expression in α-Klotho, a transmembrane protein expressed in the kidney that serves as a co-receptor for fibroblast growth factor 23 (FGF-23) (66), and a corresponding increase in FGF-23, a bone hormone that regulates phosphate homeostasis in the kidney along with active vitamin D and parathyroid hormone (67) concur to the production of secondary hyperparathyroidism (68) and hypercalcemia (69). The large use of pharmacological therapies, such as calcimimetics and antiresorptive agents, can inhibit the production of parathyroid hormone. However, these medications, together with inflammation, oxidative stress, and malnutrition, may also produce an excessive suppression of parathyroid hormone eventually leading to an adynamic bone disease, which is now the most predominant form of uremic osteodystrophy (70). In adynamic bone disease there is a reduced bone turnover, which limits the capacity of bone to release or store calcium, resulting in normo-, hypo- or hyper-calcemia (71). Thus, in dialysis patients with sarcoidosis hypercalcemia can result from many possible sources, including the underlying disease, an autonomous secondary hyperparathyroidism, an adynamic bone disease, an inappropriate use of medications such as calcium carbonate, vitamin D and derivate, or the use of high dialysate calcium concentrations (72–75). Other rare causes of hypercalcemia should also be considered, such as immobilization (76), adrenal insufficiency (77, 78), tuberculosis (79) and malignancy (80, 81). The fact that hypercalcemia may occur in anephric patients, demonstrates that calcitriol may be generated by extra-renal sources (82, 83). The development of hypercalcemia is often enhanced by the administration of vitamin D3. Some nephrologists feel that measurement of 25-OH vitamin D is sufficient to assess the deficit of Vitamin D. In sarcoidosis serum levels of 25-OH vitamin D are often low. This finding may encourage the clinician to prescribe vitamin D supplementation. However, this apparent hypovitaminosis is the result of an overproduction of 1α-hydroxylase that converts 25(OH) to the active 1,25(OH)_2_D_3_**.** Supplementation of vitamin D may aggravate hypercalcemia in these cases (84, 85). Although the most used test to measure vitamin D is based on 25-OH vitamin D, in dialysis patients we recommend to measure 1-25(OH)2 vitamin D3 to prevent iatrogenic toxicity. In a series of 101 cases of calcitriol-mediated hypercalcemia, sarcoidosis represented the most common etiology, being involved in 49% of cases (86). Whatever the cause, chronic hypercalcemia may lead to vascular calcifications which are associated with an increased risk of cardiovascular disease and mortality (87). Identifying the causes of hypercalcemia in sarcoidosis patient on dialysis is critical. In difficult cases, bone biopsy can provide important information. It may indicate or rule out the presence and severity of osteitis fibrosa, osteomalacia, malignancy, or chronic infection. Histomorphometric analysis can evaluate the bone and trabecular volumes, the ratio osteoid surface per bone surface volume and fibrous volume giving information on the presence of high turnover bone disease or adynamic kidney disease.

### Kidney transplantation

There is little information about the results of kidney transplantation in patients with sarcoidosis. From a theoretical point of view, sarcoidosis patents with ESKD should be considered good candidates to a successful kidney transplantation. Indeed, many patients are young or middle-aged adults and in advanced renal sarcoidosis, corticosteroids and other immunomodulatory treatments may attenuate disease activity. Good outcome was reported by single centers. Sarcoidosis recurred in some patients (88, 89). However, it is difficult to assess the risk of recurrence since the follow-up was too short (90–95). A few cases of *de novo* sarcoidosis developing after kidney transplantation have been described (96, 97). In a multicenter French study of 2010, 18 patients with sarcoidosis who underwent renal transplantation were reported. After a median follow-up of 42 months, patient and death-censored graft survival were 94.4% and the mean GFR was 60 ml/min per 1.73 m^2^. Recurrence of sarcoidosis occurred in 5 patients (27.5%) in median after 13 months; extra-renal involvement occurred in two patients and renal involvement in three patients (16,5%) leading to impairment of graft function. When the period between the last episode of sarcoidosis and renal transplantation was short, recurrence was more frequent (98). In summary, the available data indicate that survival and complication rates of kidney transplant in sarcoidosis are similar to those of patients undergoing transplantation for other indications. Immunosuppressive therapy including corticosteroids could control hypercalcemia. However, renal or extra renal recurrences of sarcoidosis are frequent and little information is available about the long-term results. With these limitations, kidney transplantation can be considered an acceptable treatment for sarcoidosis patients with ESKD.

## Summary and conclusion

Kidney involvement in sarcoidosis is often clinically silent but may be severe if not diagnosed or detected too late. In this descriptive review, we have reported the main case reports and retrospective studies available in literature about RRT in patients affected by renal sarcoidosis. Dialysis may be required in sarcoidosis for acute and chronic disease and both hemodialysis and peritoneal dialysis can be used, although a peritoneal sarcoidosis could be the clinical presentation of the disease and the cause of ineffective peritoneal dialysis. Based on literature data, kidney transplantation is also an accepted treatment for uremic sarcoidosis patients and complication rates of kidney transplant seem to be similar to those of patients undergoing transplantation for other indications. An early recognition of kidney diseases in sarcoidosis is crucial to reduce the risk of complications, the progression of kidney disease and to establish the prognosis.

A systematic screening of renal function including serum creatinine, urine analysis, and serum calcium levels should be part of diagnostic test in sarcoidosis patients to detect early kidney involvement and start treatment. Further larger studies are needed to confirm these clinical results and to better investigate treatment and prognosis of patients with renal sarcoidosis.

## Author contributions

All authors listed have made a substantial, direct, and intellectual contribution to the work, and approved it for publication.
